# Interviewing preschool children in Greece about their usage of mobile devices at home

**DOI:** 10.1007/s43545-022-00522-5

**Published:** 2022-09-29

**Authors:** Maria Pogiatzi, Iro Bardoutsou, Konstantinos Lavidas, Vassilis Komis

**Affiliations:** Department of Educational Sciences and Early Childhood Education, University of Patras, Patras, Greece

**Keywords:** Mobile devices, Mobile learning, Preschool children, Children’s voices, Home usage of technology

## Abstract

The time children spend with mobile devices has increased in recent years. Preschoolers’ home usage of mobile devices has mainly been investigated through parents’ perceptions. Listening to the children’s voices can help us understand this matter more directly. Therefore, in this study, a convergent mixed methods approach was employed to help us investigate the home usage of mobile devices by young children, focusing on children’s comments. The participants were 55 Greek children aged 4–6, attending two public kindergartens in Patras. Children described in detail their activities on mobile devices, through semi-structured interviews. The results indicated that the most popular activity for the children was playing digital games. The children were able to demonstrate and describe activities for educational purposes (letters, numbers, digital puzzles, documentaries, etc.) during the home usage of mobile devices. The circumstances in which they are allowed to use their mobile devices is when their parents want to keep them calm during quiet hours or in public places. Implications for introducing mobile learning activities in Greek education are discussed.

**Supplementary Information:**

The online version contains supplementary material available at 10.1007/s43545-022-00522-5.

## Introduction

The growing frequency of mobile device usage (e.g., smartphones and tablets), especially among preschool children, is related to features such as portability, lightweight, screen size, multiple representations of information and autonomy (Dashti and Yateem 2018; Nikolopoulou 2020; Wood et al. 2016). These devices are considered easy to use for children as they need only one finger to interact with the features through touch-based gestures, such as tapping, swiping, scrolling up and down, and pinching (Dashti and Yateem 2018; Neumann 2018).

The shifting to mobile technology and its usage mainly by the younger family members has been investigated by many researchers (Chaudron et al. 2015; Kabali et al. 2015; Neumann 2015; Neumann et al. 2018; Nikolopoulou 2020; Papadakis et al. 2019; Tamim et al. 2015). However, in previous research, little attention has been paid to preschool children’s usage of technology and the role of mobile technologies in education. Even though many researchers have investigated the topic of mobile technology usage at home, the interest is mainly centred on parents’ views instead of ‘children’s voices’ (Dashti and Yateem 2018; Plowman 2015). In this study, we carried out semi-structured interviews with children at age of 4–6 to investigate their use of mobile devices at home. To the best of our knowledge, the present study is one of the unique studies in terms of having data directly collected from preschool children in Greece. The inclusion of their voices and opinions provides more authenticity and reliability to our data.

The investigation of children's practices in the use of mobile devices could serve as a connection between home and school environment (Neumann et al. 2018; Palaiologou 2016). Taking into account that parents and teachers play a key role in the education and the holistic development of preschool children (cognitive, emotional, social), this investigation not only will support teachers to develop suitable learning activities with mobile devices (Dashti and Yateem 2018; Neumann et al. 2018; Plowman 2015) as well as will provide information to parents on how to support their children to effectively use mobile technology at home (Palaiologou 2016; Yuen 2011).

## Literature review

Previous research has found that the use of mobile technologies by young children is increasing every year (Chaudron et al. 2015; Dashti and Yateem 2018; Nikolopoulou 2020; Strasburger et al. 2014). Researchers have mainly examined the use of mobile devices and whether this use varies by population group, (Kabali et al. 2015; Kiliç et al. 2019; Mikelic Preradovic et al. 2016) by national contexts (Chaudron et al. 2015; Dashti and Yateem 2018), by demographic features of the users and their parents, such as age, gender, household income and educational level (Nikolopoulou 2020; Mikelic Preradovic et al. 2016; Papadakis et al. 2019). Moreover, they have investigated the parental perceptions of smart mobile technologies and how these perceptions influence the use of mobile devices by the children (Chaudron et al. 2015; Mikelic Preradovic et al. 2016; Wu et al. 2014). and the favourite activities of children on mobile devices (Dashti and Yateem 2018; Kabali et al. 2015; Kiliç et al. 2019; Mikelic Preradovic et al. 2016; Neumann 2015; Nikolopoulou 2020; Papadakis et al. 2019). Next, we present recent selected studies that investigated the mobile device usage of preschool children.

Nikolopoulou, (2020), with a sample of 100 parents with children aged 4–6 years old, explored children's home experiences of tablets and parents' views on benefits and their concerns regarding their children's use of tablets. It was shown there were many activities carried out by children, such as playing games, watching cartoons, listening to music, watching videos/YouTube and using educational apps. Activities like looking at pictures, taking pictures, and listening to audiobooks were mentioned by fewer parents. Parents stated that tablets can make learning a fun experience, and their views on the benefits of it referred to the teaching of basic technology skills, foreign languages, and math. However, parents’ main concerns included dependence, reduction of social communication and interaction as well as inappropriate content. Similarly, Papadakis et al. (2019), examined the perceptions of 293 parents regarding whether their children (aged 4–6 years old) should use smart mobile devices at home or not. Their findings indicated that smart mobile devices have become necessary in the participants’ daily life. All the participants owned at least one smart mobile device at home. 10% of the children had their own mobile device while 45% of the children were using the mobile devices of their parents. Digital games and educational apps are some of the activities that were approaching their preferences. Specific educational apps are close to the parents’ preferences because they are easy to use and they teach children math, literacy skills and language learning (mainly English), YouTube, sketching and painting apps as well as mind games such as puzzles and card games. Similar results were demonstrated in another research taken place in Croatia (Mikelic Preradovic et al. 2016) in which, 152 parents of children aged 3–7 years old supported the introduction of digital literacy education as part of the educational programme in kindergarten and express their wishes to be informed by educative workshops. The main activities on mobile devices were watching cartoons, playing games, listening to music, and interacting with educational software that teaches numbers, letters, English, days of the week, seasons, traffic signs, etc.

An investigation of children’s voices was carried out by Dashti and Yateem (2018). They interviewed 112 children aged 3–5 years, of whom 53 children live in Kuwait and 59 children live in the USA. Their main aim was to listen to young children explaining how they use their mobile devices and if there are differences in usage by their age or gender. Half of the children indicated that they used one device, either a tablet or a mobile phone, while some used both devices. The activities carried out by children mainly were to look at pictures, play games, watch videos, and draw pictures. In their research, they have found children engaged in reading e-books and they learned many things using mobile devices such as the alphabet, numbers, information about animals and songs.

As can be seen from the above, the issues related to the usage of mobile devices by preschoolers have been investigated mainly through parents’ perceptions (Chaudron et al. 2015; Mikelic Preradovic et al. 2016; Nikolopoulou 2020; Papadakis et al. 2019; Wu et al. 2014). Limited research has explored children’s usage of these devices through children’s comments (Dashti and Yateem 2018), especially in Greece. Consequently, in this study, we tried to investigate preschoolers’ home access and usage of mobile devices in Greece. Obtaining data directly from children will help us address this gap in the literature. Therefore, the corresponding research questions are:

1. How do preschoolers use mobile devices?
  (i) Do they have access to mobile devices at home?
  (ii) What is their preference between tablets and smartphones?
  (iii) What kinds of activities do preschoolers conduct on mobile devices and what is their preferred one?
2. How do preschoolers learn to use mobile devices?
3. What are the circumstances in which parents let their children use mobile devices?

## Research methodology and methods

### Participants and setting

A convergent mixed methods design was employed in this study. Both quantitative and qualitative data were collected at the same time to understand the theme (Creswell 2012). In this way, the qualitative data supports an in-depth understanding of the context. The quantification of qualitative data supports a more analytic and comprehensive image of the preschoolers’ use of mobile devices at home. Children’s descriptions of the usage of mobile devices at home were collected through semi-structured interviews (Creswell 2012). Prior to conducting the study, the researchers received approval from both the kindergarten supervisors and the Research Ethics Board (REB) designated by the University of Patras and particularly from the Department of Educational Science and Early Childhood Education. In addition, children and parents were informed by the teachers before the data collection and given the option of withdrawal at any time without any penalty. We came to an agreement with the school administrations that each semi-structured interview would last no more than 15 min. The choice of the kindergartens in the city of Patras was made by chance. In a discussion we had with the kindergarten teachers, we did not find any different approaches regarding the use of mobile devices for educational purposes in their classrooms. Both teachers stated that they only sometimes use the available computer (as mentions in the syllabus) but no mobile devices to support their lessons.

The interviews were conducted in January 2020. They were conducted in the classrooms, which is a familiar place for children, during school hours, at a suitable time and space, according to the teachers' guidelines. The sample of the study consisted of 55 Greek preschoolers, 23 children from four to five years old and 32 children from five to six years old, attending two different public kindergartens in the city of Patras, out of which 30 were male and 25 were female.

### Interview procedure

The data in this paper are drawn via semi-structured one-on-one interviews with preschool children. Semi-structured interviews are defined as having the flexibility of an unstructured interview. All questions help to extend discussions and provide opportunities for participants to best voice their experiences (Creswell 2012).

The kindergarten teachers introduced us to the participants a couple of days before the interviews, so they would feel more comfortable talking to us. As an ‘ice breaker activity’ (Chaudron et al. 2015), we spent some time playing with each participant with modelling clay, to create a child-friendly approach. The interviews were carried out in a quiet space in the classroom and were conducted by one of the authors. The duration of each interview was approximately 15 min. Having received the consent of the teacher, all of them were audio-recorded using the researcher’s mobile phone. The interviewer addressed specific questions to the participants encouraging them to express their views and practices, which were treated fairly, sensitively and without prejudice. Two devices were presented to the participants, a tablet and a smartphone, so they could recognize the devices and demonstrate to us the specific gestures while using them.

### Research instrument and data analysis

The interview protocol in this research was designed in accordance with previous studies on the topic (Dashti and Yateem 2018; Kiliç et al. 2019). Similar questions were employed, regarding the homeownership of the devices, the frequency of use and the preferable activities on them. These questions were translated into Greek using the translation/back-translation technique. Firstly, a translator converted the questions from English to Greek. Next, a second translator converted the questions back to English. After that, the two English versions of the questions were compared and points in need of changes were not observed. In the final version of the interview protocol, we added extra questions that could support the procedure. Moreover, during the interview depending on the responses of the children more details and explanations were asked (see interview protocol in Appendix). Additionally, to ensure the validity of the responses, the researcher restated some questions in a different way. Whenever there wasn’t relevancy between both responses, the researcher would ask the children for more explanations. Finally, before the actual interviews took place, the research instrument was tested on two children 5 years old. This pilot test mainly highlighted some minor changes in the wording of questions that we had to consider during the process of the interviews. All interviews were transcribed verbatim and then they were read again and again to get a sense of the whole. The qualitative data (children’s descriptions) were analysed using the thematic analysis method to develop relevant codes, categories, and themes (Saldaña 2013). The text was divided into smaller parts which were labelled by codes and then these codes were grouped into categories which created themes. The key themes were based on the four research questions as were also indicated by the thematic analysis. These themes are: (a) possession of mobile devices at home, (b) children’s preference between tablets and smartphones, (c) activities that the children perform while using devices—their favourite activity, (d) types of games, (e) types of educational use, (f) support for use of mobile devices, (g) circumstances that the children use mobile devices.

## Results

### Possession of mobile devices at home

The participants indicated that there is at least one mobile device at home, only smartphone (41.8%) and in most cases, smartphone, and tablet (58.2%). Even though their mothers' or fathers' device was used the most, the 4–5 year-old children indicated that they own a mobile device themselves, either a tablet (20.0%), a smartphone (1.8%), or both devices (3.6%).

### Children’s preference between tablets and smartphones

The children that had used both mobile devices, were asked to choose their preference between the two of them. According to their responses, more preference is shown for the tablet (65%) than the smartphone (35%).

### Activities that the children perform while using mobile devices—their favourite activities

When asked children about their mobile activities, the participants had to describe the various activities they are engaged in but also choose their favourite one among them. Table 1 shows the distribution of their responses. The participants mainly answered that they play digital games (84%), watch cartoons (74%) and draw (18%). When choosing their favourite activity, most children mention ‘Play games’ (60%), for example:

Child 1: ‘I watch videos on my grandpa’s tablet. I also like to watch Elsa’

Researcher: ‘Is there anything else that you like to do on your grandpa’s tablet?’

Child 1: ‘There is the game of unicorns. I make them look prettier.’

Researcher: ‘Do you prefer to watch Elsa or play with the unicorns on the tablet?’

Child 1: ‘To play with the unicorns’

Researcher: ‘Do you like playing games more?’

Child 1: ‘Yes’

**Table 1:** Activities on mobile devices (*N* = 55)

	Frequency	Percent
Play digital games	46	83.63%
Watch cartoons	41	74.54%
Draw	10	18.18%
Take/look at pictures	9	16.36%
Watch videos	8	14.54%
Listen to music	4	7.27%
Communication (message-phone calls)	3	5.45%

Another category is ‘watch cartoons’ (10.90%), for example:

Child 2: ‘My parents haven’t downloaded games on the smartphone, but when I use it, I watch Netflix. If there is the Greek language… First, I have to check if I can dub the video into Greek, otherwise, I cannot watch it. On Netflix, I watch a cartoon in which there is a thief.’

For the next activity, which is ‘Draw’ (7.30%) some responses are: Child 3: ‘I have downloaded a game in which you can draw with a paintbrush, whatever you want.’, ‘take/look at pictures’ (3.60%) Child 4: ‘There are no games on the mobile to play… I just take pictures, selfies, my mom taught me how to do it… I take pictures of my mom and myself’., Child 5: ‘I take record videos of my dad on the tablet while he is eating.’, ‘watch videos’ (3.60%) Child 6: ‘I watch videos with wild animals…green…dinosaurs’, ‘listen to music’ (3.60%) Child 7: ‘I watch Akadou (Greek Children Songs)’ and finally, ‘communication’ (3.60%) with family members, such as typing messages or respond to phone calls Child 8: ‘I write messages to my father on the mobile’. It is worth mentioning that some of the children preferred to use the mobile devices we offered them to demonstrate their usage.

### Types of games

The children in this research describe that they mainly use mobile devices for playing games. Examples demonstrating the use of mobile devices for digital games are: Child 1: ‘I like the smartphone the most because I can download games on it, like Lego Ninjago. I have watched the videos to understand the rules of the game.’, Child 2: ‘My brother has downloaded games on the mobile so we can play, there is one with a skateboard.’, Child 3: ‘There is this game in which I have to smash all kinds of fruits’, Child 4: ‘I have downloaded a game in which there are some girls and I change the way they look. I can choose dresses, makeup and shoes, there are also purses’., Child 5: ‘I like to play Fortnite’., Child 6: ‘My dad has this game with Tom, the little cat so I take care of him’. It is worth mentioning that some of the children demonstrated how they use their mobile devices. Table 2 shows the distribution of digital games as mentioned by the participants.

**Table 2:** Digital games on mobile devices

Digital games	Frequency	Percent
Simulation games	10	18.2%
Action games	9	16.4%
Action games (platform)	6	10.9%
Racing games	5	9.1%
Strategy games	5	9.1%
Racing games/action games	1	1.8%
No digital games	19	34.5%
Total	55	100.0%

### Types of educational use

Intensifying the discussion on the activities carried out by the mobile devices, half of the participants responded positively about their educational use at home. Table 3 shows the distribution of their answers to the types of educational use. Examples of responses included the following:

Child 1: ‘I like playing with music, creating melodies…and I have a game in which I learn how to tell the numbers in English.

Researcher: ‘In English?’

Child 1: ‘Yes. And in Spanish!’,

**Table 3:** Types of Educational use on mobile devices at home

	Frequency	Percent
Letters/numbers	7	12.7%
Only letters	10	18.2%
Only numbers	4	7.3%
Educative videos/puzzle/letters	3	5.4%
Only puzzle	2	3.6%
Foreign languages/create melodies	1	1.8%
None	28	50.9%
Total	55	100.0%

Other children stated: Child 2: ‘I share the tablet with my brother. I watch some videos… I do not remember… playmobile and documentaries about birds. Mom searches it for me’, Child 3: ‘I play a game with numbers…I have to put them in the correct order’, Child 4: ‘I play with shapes. I made a rectangle’, Child 5: ‘I watch documentaries. I watched a documentary with birds’. Another example of educational use is the following:

Researcher: ‘Is there anything else that you enjoy using the tablet?

Child 6: ‘I like to play this game of writing words’

Researcher: ‘Are there letters?’

Child 6: ‘The letters are down here. I use them to write words.’

Researcher: ‘But how are you doing this?’

Child 6: ‘The letters are down here, I can choose them, and then I lift them here but in the right order, in their right place. I also do it with numbers’

Researcher: ‘How are you doing this;’

Child 6: ‘They are down here mixed up. I touch them and I lift them in the right order.

In some cases, children seemed to not use their mobile devices for educational purposes, for example, a child mentioned*:* ‘This is for older children’.

### Support for the use of mobile devices

Few of the participants (20%) indicated that they receive help when interacting with mobile devices mainly mentioning their parents and their siblings. For example: Child 1: ‘There is this game where I must find clues, then make puzzles and find the passwords. Small notes with passwords. My dad helps me to make it’, Child 2: ‘My dad showed me a little (how can I use the tablet)’, Child 3: ‘My mom helps me to play Fortnite’, Child 4: ‘I took selfies. My mom taught me. My mom, my godmother and my godfather (taught me)’, Child 5: ‘My dad touches a button (οn the tablet) to open, and after I touch the buttons to play’.

### Circumstances that the children use mobile devices

The circumstances under which parents let their children use the devices are presented in Table 4. Children cannot fully comprehend the concept of time duration (Droit-Volet et al. 2006; Levin 1977), so these questions were to determine the frequency approximately. Representative extracts that illustrate the usage pattern are the following: Child 1: ‘In the afternoons when we go to eat at my grandmother’s place with mom and dad.’, Child 2: ‘My dad gives me the tablet when I go to his shop…because he must have his smartphone and watch something else’, Child 3: ‘I play with my tablet in the afternoons because (in the afternoons) not many friends come at my home’, Child 4: ‘They allow me to play when I finish my food in the afternoons’.

Child 5: ‘When we are in the village, at the square and home.’

Researcher: ‘Why do they give it to you then?’

Child 5: ‘Because there isn’t anyone else around to play and so they give it to me.’

Researcher: ‘Why do they allow him to use the device at that time’

Child 6: ‘My mom allows me to use it at night because she is doing house chores at that time’,

**Table 4:** Use of mobile devices at home throughout the day

	Frequency	Percent
Public peace hours/afternoon/night	38	69.1%
Whenever I want	5	9.1%
No permission	3	5.5%
The weekends	2	3.6%
Public places	2	3.6%
Before school	2	3.6%
Reward/punishment system	2	3.6%
Restrictive use	1	1.8%
Total	55	100.0%

while another participant stated (Child 7): ‘In the afternoon…. Because they don’t want me to fall asleep’. Furthermore, it seems that digital devices are a part of the punishment/reward system in families:

Child 8: ‘My grandfather has a tablet, that’s why I can recognize it. My parents have smartphones, but I cannot find them. I look for them, but I cannot find them. My grandfather allows me to use it when I behave. When I am a good child.’

and in some cases, children do not have permission to use their mobile devices at all:

Child 9: ‘I’ve downloaded some games on the smartphone. They don’t allow me to use it every day, but I use it secretly anyway’.

## Discussion

In this study, we investigated children’s voices in using mobile devices at home. Regarding the first research question, as several researchers have indicated (Chaudron et al. 2015; Kiliç et al. 2019; Neumann 2015; Papadakis et al. 2019), this study similarly found that the most owned and favourite mobile device is the tablet. Teachers and parents could make good use of this preference in the classroom to motivate the children in the educational process. A small percentage (7.2%) of the participants reported owning a fake mobile device. This finding indicates a positive, yet hesitant behaviour on behalf of the parents regarding the use of mobile devices.

In relation to the first research question, the participants in this research engage in a range of activities, but they mainly use mobile devices for entertainment (Chaudron et al. 2015; Dashti and Yateem 2018; Mikelic Preradovic et al. 2016; Neumann 2018; Nikolopoulou 2020). A rather surprising outcome of this research is that during the interviews, a significant amount of the children was eager to operate the devices, while their gestures were consistent with their previous comments. This evidence supports that the information shared with the researchers was extracted from reality and not from their imagination. Furthermore, it seems that they are quite familiar with mobile technology. The children are particularly fond of playful apps (e.g., digital games) when using mobile devices. The children in this research chose ‘digital games’ over ‘watching cartoons’ as their favourite activity by far (Dashti and Yateem 2018). This implies that the usage of mobile devices is not entirely passive and that there is a high level of cognitive skills that are carried out during the interactive play with the apps. Additionally, the results indicated that the most popular types of gaming apps that were mentioned by the participants are: Action games (e.g. Fruit Ninja), Platform games (e.g. Super Mario, Skater Kid), Shooter games, endless runner games (e.g. Subway surfers, PJ Masks Starlight Sprint), Fighting games, Racing games, Strategy games (e.g. Fortnite) and finally Simulation games (Pet raising—e.g. My talking Tom, controlling the appearance of avatars and artificial lives—e.g. Barbie games).

Half of the preschoolers in this research described that they use the tablet or the smartphone even for educational purposes. This finding is consistent with other researchers on this topic (Dashti and Yateem 2018; Chaudron et al. 2015; Nikolopoulou 2020; Palaiologou 2016). The children indicated that they learned many things by interacting with playful applications with letters, numbers, solving puzzles, watching educative videos on YouTube, colouring and in just one case, creating music. Teachers could utilize this evidence in formal education for optimal learning outcomes. They could design proper activities in which preschoolers would use mobile devices, combining in this way children’s entertainment and education. Mobile devices can be a teaching tool in the teacher’s or parent’s hands, as the current experience of distance learning, due to the Covid-19 pandemic has indicated (Apostolou and Lavidas 2021; Lavidas et al. 2022). Some training for parents and teachers on the educational use of mobile devices may be necessary. Additionally, the game designers could build more educational games for preschoolers.

Concerning the second research question, it is worth mentioning that some children described that they receive help while using mobile devices, mainly from their siblings or their parents. Especially, when mobile devices are being used for educational purposes, the parents are instructing their children on how to use them. Overall, it seems that most of the children are capable enough to interact or experiment-play with the devices on their own. This finding is important to know, as the parents have to be aware of how preschoolers’ can use their mobile devices when they are alone. Also, parents could select well-designed and age-appropriate activities for their children to increase their positive effects in contrast to the negative.

Finally, in relation to the third research question regarding the circumstances in which parents let their children use the devices, they seem to maintain the role of “filling leisure time” (Chaudron et al. 2015). The most presented usage pattern is during the quiet hours, which are after lunch (afternoon)—or during the night, before bedtime (Chaudron et al. 2015; Kabali et al. 2015). This pattern indicates that parents use media as pacifiers, giving devices to their children to keep them calm during quiet hours, in public places or when they are busy while performing various daily tasks (Chaudron et al. 2015; Chen et al. 2019; Dashti and Yateem 2018; Kabali et al. 2015; Kiliç et al. 2019; Neumann et al. 2018).

## Implications and limitations

This research contributes to identifying the ways in which the power of mobile technology will support the advancement of education for children and therefore provides assistance to teachers, developers, and researchers to develop suitable applications and learning activities supported by mobile devices. Having received this feedback from preschool children, it seems that designing digital educational games is one of the ways to encourage motivation in either formal or informal settings while it promotes active engagement than passive learning experiences (Kolovou et al. 2021; Lieberman et al. 2009; Mccarthy et al. 2013; Strasburger et al. 2014). Active engagement in the learning process would be the result of integrating the favourite educational digital games of children that connect home and school environments (Mccarthy et al. 2013; Yuen 2011).

The results acquired from this study are valuable for further consideration, particularly by early childhood researchers. For example, to further investigate the way in which preschool children acquire operation skills before or while using mobile devices. Adding parents’ descriptions simultaneously with children’s voices would be useful. Considering that families have become increasingly reliant on screens, parents and children are in need of recommendations to help them coexist with mobile devices in a healthy and productive way (Neumann et al. 2018; Papadakis et al. 2019).

Finally, the results of the present study should be considered in light of certain limitations. An issue that needs to be paid attention to is that during the interview with 4–6 years old children their responses might get affected as they can get intimidated by the process (Creswell 2012). Moreover, the limited sample size prevents the generalization of the findings to the general population. Therefore, in future research larger sample should be investigated.

## Conclusion

The overall findings of the interviews with preschool children reveal the frequent and independent use of mobile devices. Considering this, parents need to guide and supervise young children on how to appropriately use their mobile devices and even co-viewing, when possible, to reduce the negative consequences of excessive screen use. The most owned and preferred device is the tablet which is used for entrainment purposes during leisure time. The most preferred activity (e.g., digital games) indicates a more interactive use of the devices. A surprising finding of the interviews is that over half of the participants use smartphones and tablets at home in activities with an educational context. Children receive help while using mobile devices, mainly from their siblings or their parents. The circumstances in which children are allowed to use their mobile devices is when their parents want to keep them calm during quiet hours or in public places. This finding indicates that the learning benefits of smartphone technology cannot be ignored. Parents and educators should model a safe and responsible mobile phone use for young children’s development in a digital world. Furthermore, the findings highlight the need for developing and accessing educational apps on mobile devices for the target audience of preschool children.

## Supplementary Information

Below is the link to the electronic supplementary material.

Supplementary file1 (DOCX 32 kb)

## Data Availability

The datasets generated during and analyzed during the current study are available from the corresponding author on reasonable request.
