# Supplementary material for: Interviewing preschool children in Greece about their usage of mobile devices at home
Source: SN Soc Sci. 2022 Sep 29;2(10):215. doi: 10.1007/s43545-022-00522-5 (PMC9520108; doi:10.1007/s43545-022-00522-5)
Supplement: Supplementary file 1 — Supplementary file1 (DOCX 32 kb) [file 43545_2022_522_MOESM1_ESM.docx]

# Appendix. Interview Protocol

‘Do you have a mobile device at home?’ (If the children answer yes) ‘Does it belong to you or your parents?’

‘Do you have your own mobile device at home?’. (If the children answer yes) ‘Which?’.

If the participants owned both mobile devices at home. ‘Do you prefer the tablet or the smartphone?’, ‘Why?’

The activities in which children participated using mobile devices were determined by the following questions:

‘What’s your favourite activity while using the tablet or the smartphone?’ for example, ‘Do you prefer to watch cartoons or to play the game that you mentioned before?’.

‘Did you ever try to use the mobile device to play with letters? Describe it’,

‘Did you try to use the mobile device to play with numbers? Describe it’.

The circumstances under which parents let their children use the devices were indicated by the following questions:

‘Do your parents allow you to use the tablet/smartphone every day?’

‘Which moment of the day, night or day? Before or after school?’

‘Why do they let you use it at that time?’.
